# A Unified Discussion on the Concept of Score Functions Used in the Context of Nonparametric Linkage Analysis

**Published:** 2008-02-14

**Authors:** Lars Ängquist

**Affiliations:** Centre for Mathematical Sciences, Department of Mathematical Statistics, Lund University, Lund, Sweden

**Keywords:** nonparametric linkage analysis, allele sharing, genetic disease models, inheritance vectors, score functions, families of score function definitions, genetic models, NCP-optimality, IBD-sharing structures, equivalence of score functionse

## Abstract

In this article we try to discuss nonparametric linkage (NPL) score functions within a broad and quite general framework. The main focus of the paper is the structure, derivation principles and interpretations of the score function entity itself. We define and discuss several families of one-locus score function definitions, i.e. the implicit, explicit and optimal ones. Some generalizations and comments to the two-locus, unconditional and conditional, cases are included as well. Although this article mainly aims at serving as an overview, where the concept of score functions are put into a covering context, we generalize the noncentrality parameter (NCP) optimal score functions in Ängquist et al. (2007) to facilitate—through weighting—for incorporation of several plausible distinct genetic models. Since the genetic model itself most oftenly is to some extent unknown this facilitates weaker prior assumptions with respect to plausible true disease models without loosing the property of NCP-optimality.

Moreover, we discuss general assumptions and properties of score functions in the above sense. For instance, the concept of identical by descent (IBD) sharing structures and score function equivalence are discussed in some detail.

## 1 Introduction

**Figure 1:**
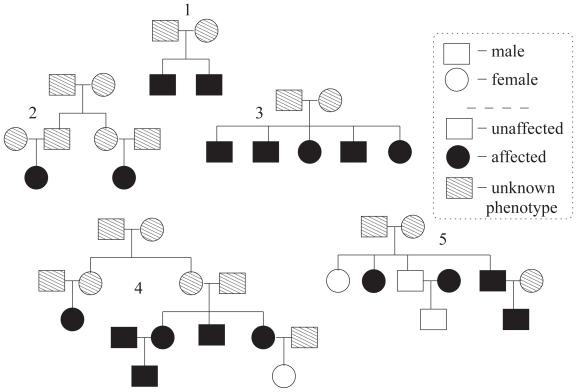
A pedigree set example consisting of 5 distinct pedigrees of different structures and phenotype settings.

In *linkage analysis* (Ott, 1999) or, in a wider sense, *gene mapping* (Haines and Pericak-Vance, 2006; Siegmund and Yakir, 2007) one searches for disease loci along genetic regions of interest; in other words, through what we refer to as a *genome*. This is done by observing so called *genotypes* and *phenotypes* of a *pedigree set*, i.e. a set of multigenerational families, throughout the genome. The rationale for doing this is that, at a disease locus, the genotypes and phenotypes should generally show correlation of some strength on the individual level within the pedigree, where the actual strength depends on the structure of disease, i.e. the so called *genetic model*. Observed present correlations, measured through some kind of test statistic, suggests localizations of loci corresponding to underlying disease genes or, at least, it narrows down the interesting genome regions to neighbourhoods of the findings. The amount of trust put into such loci actually being disease-related are generally evaluated, in a standard sense, through statistical significance calculations; preferably corrected for the multiple testing throughout the genome. An example of a small pedigree set is given in Figure 1. To further reduce the size of a plausible region for an interesting disease finding, i.e. to use a fine-mapping technique, one may, for instance, use methods from the toolbox of *association analysis* (see Balding, 2006).

### 1.1 Basic notation and concepts

In practise the genotypes are observed as well-defined *allelic types* at polymorphic marker loci located along the genome of interest. Vaguely speaking, a marker locus might be seen as an, in some sense, observable short chromosomal segment and it is polymorphic if several types of genetic observations are possible, in the underlying population, with respect to this segment. Hence polymorphic markers correspond to genetic variation in the population.

#### Example 1 (Alleles and genotypes)

Consider a situation where we have a polymorphic locus with respect to three distinct possible allelic types-outcomes A, B and C within the population. Hence, at this locus, a specific individual will have any of the six consistent unordered genotypes; AA, AB, AC, BB, BC and CC, with certain probabilities jointly summing to one. For more information on, for instance, alleles, genotypes and genetic markers cf. Strachan and Read (2003).

As a restriction or application, in *nonparametric linkage (NPL) analysis* (Whittemore and Halpern, 1994; Kruglyak et al. 1996; Ängquist, 2007) one searches for genetic linkage between disease and marker locus by observing and analyzing marker genotype data, without explicitly assuming a known genetic disease model. As noted above the linkage analysis approach may somewhat vaguely be described as analyzing the amount of dependence or correlation between genotypes and phenotypes among the observable individuals in the data set at hand, and hence in the nonparametric case one does not incorporate information on any disease loci in the standard analysis or search. Most oftenly such data is then taken to be representative of a homogeneous underlying population. Note that generally the phenotypes are assumed to be qualitative in the working form of indicators of *disease status*.

In this context the prime quantity of central importance to the actual statistical analysis-procedure is the process of *inheritance of alleles*. Each individual inherits two alleles, i.e. a genotype, at each chromosomal locus; one from the father and one from the mother. The inherited alleles themselves originates from either the corresponding grandfather or the grandmother and this leads to the following statement:

For a single pedigree, at locus *x*, the inheritance process may be totally described by the binary—zero-one—*inheritance vector* (Donnelly, 1983),

(1) $$v(x)=(p_{1},m_{1},p_{2},m_{2},\ldots ,p_{n-f},m_{n-f})$$

where *p_i_* and *m_i_* correspond to the *i*th nonfounder’s paternal and maternal allele respectively, i.e. each value is connected to one of the *m* = 2(*n* − *f*) specific meioses.^1^ Note that, for instance, one may in practise let 0 and 1 correspond to inheriting grandpaternal and grandmaternal alleles respectively.

#### Example 2 (Founders and nonfounders)

**Figure 2:**
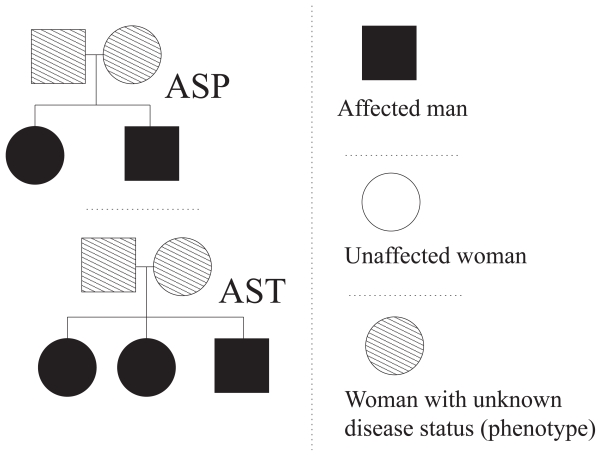
The pedigree structures corresponding to affected sib-pair (ASP) and affected sib-trio (AST) pedigrees.

Consider the pedigrees in Figure 2. In both cases the parents constitutes the set of founders, whereas the siblings are the nonfounders.

In the same manner as (1), but somewhat more obscure, one may summarize inheritance through *IBD-sharing structures*, where IBD means *identical-by-descent*. Two alleles are IBD if they are both ancestrally inherited from the same unique founder allele^2^ with respect to the corresponding pedigree. Basically, forming IBD-sharing structures means grouping the elements of the set of all the 2*^m^* possible inheritance vectors, 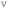, according to some pedigree-relational symmetry rules, into distinct IBD-groups. Such symmetry rules are, at least in principle, to some extent subjective. A commonly accepted example is that inheritance vectors will fall into the same group if they correspond to, i.e. one gets the same inheritance structure, permuting the inheritance of two siblings (with corresponding offsprings). Most oftenly, this is accepted even if the siblings being of distinct sexes. For more information, see Example 3 and Appendix A.

To numerically facilitate analysis of inheritance and phenotype-genotype dependence one may introduce a *score function*. Expressed in general terms this is just a function *S* giving a (numerical) score *S*(*v*) to each possible inheritance vector *v* ∈ 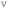, i.e. it serves as a representation of the quantification of phenotype-genotype correlation.^3^ Normally one searches for inheritance-wise deviations in the form of increased allele-sharing among affecteds,^4^ since this indicates presence of genetic linkage between the marker and disease loci. As a consequence one aims at giving inheritance vectors consistent with such increased sharing high scores. On the other hand vectors being non-consistent, in this sense, are then given low scores.

#### Assumption 1

We assume that score functions are invariant within IBD-sharing structures. Explicitly, this implies that each inheritance vector v corresponding to a specific structure A produces the same output (score), i.e.

$$S(v)=S(w);\forall v,w\in V_{A},$$

*where* 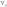 *is the equivalence class including all inheritance vectors corresponding to structure A.*

Hence considering a pedigree with *m* corresponding meioses leads to 2*^m^* possible scores,

(2) $$S=\{S(v_{1}),S(v_{2}),\ldots ,S(v_{2^{m}}),\}=\{s_{1},s_{2},\ldots ,s_{2^{m}}\},$$

assuming some order of inheritance vectors.^5^ In this setting some scores will according to symmetry, and in some cases by—explicitly or implicitly—definition, be numerically equal. Using the context of IBD-sharing structures one may reformulate (2) as

(3) $$S=\{s_{1},s_{2},\ldots ,s_{n}\},$$

where the index corresponds to the by-score-ordered set of IBD-sharing structures, i.e. a natural restriction (order) is given by assuming *s*_1_ < *s*_2_ < … < *s_n_*. Quite naturally one may note that *n* ≤ 2*^m^*^−^*^f^*.

#### Remark 1

*In fact one may instantly note that n* ≤ 2*^m−f^, where f is the number of founders, which follows from what is generally referred to as ‘founder couple reduction’ (**Kruglyak* et al. *1996**;* *Gudbjartsson* et al. *2000**). This is an inheritance symmetry property originating from the uncertainty of founder phases (ambiguity of inheritance vector interpretation)*.

#### Example 3 (Founder couple reduction)

*For an affected sib-pair (ASP), see Figure 2, one may illustrate Remark 1 through the following example: Let the parents have genotypes {A, B} and {C, D}. Oftenly the inheritance vector is defined with each position corresponding to a well-defined paternal or maternal allele of a specified nonfounder; see (1). This is then also reflected in the ordering of the alleles (including the founder alleles) in the sense that, for instance, the left allele (A and C) correspond to paternal inheritance and the right allele (B and D) to maternal inheritance. Since most likely*^6^ *the ordering (phases) of the founder alleles is unknown we, in these cases, do not really know which of the following ordered founder-genotypes that is truth-valid:*

AB/CD,AB/DC,BA/CD,BA/DC.

The implication of this is that all inheritance vectors related through transformations between these founder-genotypes are inheritance-wise evidentially equivalent. (Hence giving rise to equivalent IBD-sharing structures; see Appendix A.)

For further information on equivalent IBD-sharing structures consider Appendix A.

### 1.2 Aims and scope

Our primary goal with this paper is to, in such a generally accessible way as possible, formalize and discuss the structure of nonparametric linkage score functions. Oftenly, in published works, these functions are either directly applied using some of the standard instances or derived in an ad hoc or highly theoretical, or non-intuitive, fashion.

Having this in mind, the text to follow is not a complete summary of suggested and published score function variants, or the most theoretical exposition out there. Rather, it aims at being a review-like overview discussing the underlying structure, contexts of derivations and interpretations (and to some extent performance) of certain families of NPL score functions.

In *Section 2* three distinct such families—the implicit, the explicit and the optimality-based one—are introduced and discussed, whereas *Section 3* gives a new generalization of an existing optimality-based function. A small simulation study with respect to five distinct score functions of various types is performed in *Section 4*. The two appendices, *Appendix A* and *Appendix B*, discusses equivalence-properties with respect to structure and standardization of score functions respectively.

## 2 Approaches to Score Function Definitions

For an underlying disease to be genetically inheritable, i.e. to include a *genetic component*, some kind of correlation between the phenotype and the disease genotypes must exist. This is usually described by means of a *genetic model λ*. One may note that *λ* usually, at least to some extent, is unknown so, if needed, it is estimated prior to analysis using so called *segregation analysis* (Khoury et al. 1993; Haines and Pericak-Vance, 2006). The complete, possibly multilocus, genetic model may be summarized as,

(4) λ=(p,f,l),

where *p* is the set of *disease allele frequencies*, *f* is the set of *penetrance values*, describing the link between phenotypes and disease genotypes, and *l* defines the *disease loci positions*.

Now, to define a score function one basically has to instantiate the numerical scores corresponding to (2) or (3). This may be done in several distinct ways, which is furtherly discussed below. What truly is the core question with respect to such definitions is the evidential performance of the corresponding score function. (Most likely in the form of *statistical power* calculations.) One may note that the relative performance of different score functions depends on the underlying genetic model *λ* and the combined present pedigree-structure of the pedigree set.

A score function performing well under a wide range of different *λ* ∈ Λ, where Λ is the set of all possible disease models, is termed a *robust* score function. The best score function with respect to a criterion *C* and disease model *λ* is called an *optimal* score function *S*_opt_ = *S^C^* (*v*|*λ*).

### 2.1 Implicitly defined versions

Vaguely speaking, as noted above, at a true disease locus, the IBD-sharing within phenotypes should be expected to increase. This makes it possible to define functions, depending on pedigree IBD-sharing only, meeting this requirement (property). Since such functions implicitly instantiate (2) and (3) through the higher-level sharing-based function definition we call them *implicitly defined score functions*. Next, we will note on two distinct such definitions.

#### 2.1.1 Traditional score functions

Firstly, *S*_pairs_ (Weeks and Lange, 1988) is based on IBD-sharing among all pairs of affected individuals in the pedigree,

(5) $$S_{\text{pairs}}(v)=\sum_{(a_{i},a_{j})\in A}\text{IBD}(a_{i},a_{j}),$$

where *i* < *j*, 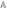 is the set of affecteds in the pedigree^7^ and IBD(*x, y*) is the number of alleles shared IBD between individuals *x* and *y*.

Secondly, *S*_all_ (Whittemore and Halpern, 1994) is based on the simultaneous IBD-sharing among all the affecteds in the pedigree,

(6) $$S_{\text{all}}(v)=\frac{1}{2^{|A|}}\sum_{h\in ℍ}\prod_{i=1}^{2f}b_{i}(h)!,$$

where | 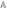
| is the number of affecteds, ℍ is a set containing the elements corresponding to all ways of selecting one allele from each affected, 2*f* is the number of founder alleles in the pedigree and *b_i_* (*h*) is the number of times the *i*th founder allele is present in selection *h* ∈ ℍ.^8^

##### Example 4 (Score function *S*_all_)

*For an ASP (Fig. 2) one may examplify (6) as follows: Let the parents have genotypes {A, B} and {C, D}. Then, if the affected siblings inherit {A, C} and {A, D} respectively we have the following h-selection possibilities (∀h* ∈ ℍ*):*

$$h_{1}=\{A,A\};h_{2}=\{A,D\};h_{3}=\{C,A\};h_{4}=\{C,D\},$$

*where for instance, treating A as the* 1*st founder allele*, Π*_i_*_=1_^4^ *b_i_* (*h*_1_) =2!0!0!0!=2.

##### Remark 2

Both (5) and (6) give high (low) scores to excess (low) IBD-sharing. The difference lies in that the latter one, relatively seen, upweight increased sharing of specific founder alleles within large groups of individuals, thus reflecting a higher degree of belief in such inheritance evidence.

#### 2.1.2 Extended score functions

Both functions (5) and (6) are defined, given the inheritance vector *v*, with respect to the set of affecteds 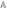 only, which might be notationally pointed out as *S*(*v*) = *S*(*v* | 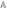). Henceforth we refer to such score functions as *traditional* score functions. In fact a vast majority of the most commonly used functions are of this kind.

In Ängquist (2006) several extensions to traditional score functions are given. Now, assume a traditional instance *S* and let *S*′ denote a corresponding *extended* version. A first-level extension is to combine information from both phenotype groups (affecteds as well as unaffecteds) through

(7) $$S^{\prime }=S^{\prime }(v)=S^{\prime }(v|A\cup UA)=S(v|A)+S(v|UA).$$

This aims at additionally searching for unusual IBD-sharing within the set of unaffecteds 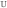 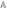. Note that *S*(*v* | 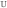 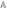) in practise means, given inheritance vector *v*, applying the traditional score function *S* to the same pedigree set, in the standard way using the same function-definition, but with phenotypes interchanged between affecteds and unaffecteds.^9^

##### Example 5 (Extended score functions; phenotype-switching)

**Figure 3:**
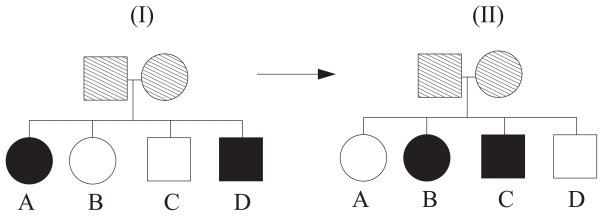
A pedigree consisting of 4 siblings (two affecteds, two unaffecteds). The two distinct cases (left to right) display the corresponding phenotype-switching process involved in the definition of extended score functions.

*Consider the pedigree consisting of two parents (unknown phenotypes) and four siblings (A, B, C and D) in Figure 3. When calculating S(v*| 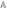
*) this is done with respect to Siblings A and D. After the phenotype-switching process displayed in Figure 3, S(v* | 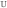 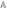
*) is calculated using Siblings B and C. Note that the actual score function algorithm, for instance underlying (5) or (6), is the same in both cases.*

A second-order extension may be formulated as

(8) $$\begin{array}{l} S^{\prime }=S^{\prime }(v)=S^{\prime }(v|A\cup AU\cup UP)\\ =[S(v|A)-S(v|A\cup AU\cup UP)\\ +[S(v|UA)-S(v|A\cup UA\cup UP)]\\ =S(v|A)+S(v|UA)\\ -2S(v|A\cup UA\cup UP), \end{array}$$

where 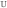
ℙ denotes the set of individuals with unknown phenotype. Here one additionally corrects for the *overall* sharing within the pedigree, i.e. it compares the IBD-sharing (through the traditional function *S*) within phenotype-groups to what is jointly given on the pedigree-level.

##### Remark 3

*An intuitive critiscism to extensions as (7) and (8) might be that unaffecteds is not to the same extent as affecteds a secure (final) phenotype, since in time such individuals might turn into affecteds.*^10^ *However, this could be solved by letting ambiguous cases, according to some criterion, being labelled as having affection status unknown, i.e. as* 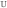
ℙ*-individuals. Further, given a well-defined probability model for (possible) affection time one may weight the analysis (scores) with respect to this model*.

##### Remark 4

Another objection against usage of unaffecteds in this way may be raised if a disease is not purely caused by gene mutations, but rather through a combination of genetic and environmental factors. In this case the unaffecteds in the pedigree are not obviously good representatives of the normal population in terms of genetic composition. It is then logically possible that such unaffecteds still share a common genetic background with the affected relatives, but lack certain environmental factors; or lack some trigger events in their health history. Hence, their role in gene mapping is in this case of secondary importance.

### 2.2 Explicitly defined versions

It is perfectly possible not to use a closed definition or high-level algorithm when calculating the vector of scores constituting the corresponding score function. We refer to such cases as *explicitly defined score functions*.

The construction of an explicit score function reduces to (explicitly) distributing scores to all present IBD-sharing structures, thus reflecting numerically the assumed connection between these sharing structures and evidence for a present disease locus. For instance, such an approach might be interesting if one can show by some real examples, or a priori assume, that certain combination of inheritance vector states are impossible or unlikely.

#### Example 6 (Explicit ASP-definition)

*Once more, consider an ASP. Here three IBD-sharing structures (with scores s*_1_*, s*_2_ *and s*_3_*) are possible corresponding to the sib-pair sharing 0, 1 and 2 alleles IBD respectively. Arbitrarily fixing s*_1_ *and s*_3_ *with s*_1_ < *s*_3_ *the closure of an explicit definition is reflected by the choice of s*_2_ *with the restriction of s*_1_ ≤ *s*_2_ ≤ *s*_3_*; see Section 2.4 and Appendix B.*

Explicit definitions, so to speak, implicitly make some (though quite vague) assumptions on the type of underlying disease structure. In this sense they are more strongly directed towards certain disease models than implicit definitions, but much less so than the family of definitions described below in Section 2.3. There explicit assumptions on true (plausible) genetic disease models *λ* under corresponding alternative hypotheses *H*_1_ are made.

### 2.3 Optimality defined versions

If having an explicit algorithm (as for implicitly defined versions) but where this algorithm is formulated with respect to, in some sense, an optimality criterion *C*, we say that we deal with *C-optimal* score functions.

Given a disease model *λ*, define the expected score at the disease locus under this model as

(9) $$E(S|\lambda )=\sum_{w\in V}S(w)P(w|\lambda ),$$

where *P*(*w*|*λ*) is the *inheritance distribution* under disease model *λ*. The expected value in (9) is referred to as the *noncentrality parameter (NCP)*. It is showed in Ängquist et al. (2007) (based on results given in Hössjer, 2005) that optimal score functions with respect to (maximization of) NCPs may be expressed as

(10) $$S(w)\propto P(w|\lambda )-2^{-m},$$

with *m* equaling the number of meioses. This approach might be interpreted as basing the scores on the difference between inheritance vector-probabilities under the null and alternative hypothesis in all cases.^11^ The rationale for being interested in NCPs are that this concept is closely linked, but not equivalent, to statistical power (Feingold et al. 1993).

Hence one may note that the optimal score function (10) depends on the true genetic model and should be interpreted as, in this sense, the best possible result that the investigator might expect when the genetic model is correctly specified. In practice though, the genetic model is often unknown. Then in a natural way, for each choice of score function and for a range of different genetic models, (10) facilitates comparisons with optimality, leading to a quantification of the apparent loss of information. The optimal score function might also serve as a form of explicit score function with respect to certain assumptions or prior information.

Further, in Hössjer (2003) *locally most powerful* tests are outlined using specific parametric models (in the form of exponential expansions) for the inheritance distribution under alternatives. Consider also the discussions in Whittemore (1996), Kong and Cox (1997), Nicolae (1999) and McPeek (1999).

### 2.4 Equivalent score functions

As a way of enhancing interpretation one usually uses *standardized* versions of the score functions. Standardization is performed through

(11) $$S(v)\leftarrow \left[\frac{S(v)-\mu }{\sigma }\right],$$

where, for a pedigree with *m* meioses,

$$\{\begin{array}{l} \mu =E(S|H_{0})=\sum_{i}2^{-m}S(w_{i})\\ \sigma^{2}=V(S|H_{0})=\sum_{i}2^{-m}S{\left(w_{i}\right)}^{2}-\mu^{2} \end{array},$$

are the mean and variance of *S* prior to standardization; under the null hypothesis *H*_0_ of no linkage and where summation is over all the 2*^m^* distinct elements *w* ∈ 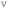.^12^

#### Remark 5

Note that S on the right-hand side in (11) is referred to as an ‘unstandardized’ score function, whereas S on the left-hand side is a ‘standardized’ score function.

Equipped with the concept of standardization one may define *equivalent* (unstandardized) score functions. In order to define this concept in a clear and straighforward manner we need the following additional assumption.

#### Assumption 2

*We assume that there is a general agreement on the order of the IBD-sharing structures, i.e. that s_i_ (*∀*i) in (3) correspond to the same structure regardless of which score function you choose.*

If two unstandardized score functions through standardization are transformed to equal^13^ standardized score functions they are referred to as being equivalent. For more detailed information and corresponding equivalence-criterions, see Appendix B.

#### Example 7 (Equivalence of *S*_pairs_ and *S*_all_ for ASPs)

*For an ASP the score functions S_pairs_ and S_all_, defined in (5) and (6) respectively, are equivalent. This follows since they both lead to the distinct standardized numerical scores* $\left\{-\sqrt{2},0,\sqrt{2}\right\}$*. Adopting the approach in (2) these scores correspond to, in turn, 4, 8 and 4 distinct inheritance vectors related to the ASP sharing 0, 1 and 2 alleles IBD respectively. (Here we have m =* 4 *meioses and* 2^*m*^ = 16 *unique inheritance vectors.) Alternatively, one may use (3) where* 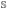 *only contain these three scores (structures), which are then attained with probabilities 0.25, 0.50 and 0.25 respectively under H*_0_.

One may also note that actual numerical standardized scores corresponding to a specific score function (or several equivalent ones) are dependent on the score distribution *P*(*s*|*H*_0_) under the null hypothesis *H*_0_, which is given by the actual pedigree structure and phenotype setting.^14^

### 2.5 Real studies and data

Note that throughout this article we try to discuss score functions without explicitly mentioning the actual test statistics they are used in connection with when facing real and imperfect marker data MD.^15^

An exception is the use of standardization through (11) which implicitly refer to the practise of the ‘NPL score’ test statistic (Kruglyak et al. 1996; Ängquist, 2007).

(12) Z(x)=E(S[v(x)]∣MD),

where the expected value, at locus *x*, is taken over *P* [*v*(*x*)|MD] which is the inheritance distribution given the observed marker data.^16^

Given imperfect data the variance of the NPL score *V*(*Z*) ≤ *V*(*S*), hence if decreasing leading to conservative procedures assuming *V*(*Z*) = *V*(*S*) = 1. In order to increase the actual variance in data, hence reducing the conservativeness, one usually bases real studies on so called *multipoint analysis*, where all inheritance information from the surrounding chromosome is used when calculating the inheritance distribution at a locus. Here the calculations are preferably performed using *Hidden Markov Models (HMMs)* through the *Lander-Green-Kruglyak algorithm*; see Lander and Green (1987), Kruglyak et al. (1995) and the expositional review in Ziegler and Koenig (2006).^17^ Actually, the complete marker data assumption seems fairly realistic when all pedigree members are genotyped with a density of SNP markers of at least, say, 0.1 cM.

Replacing *σ*^2^ in (11) with *V*(*Z*) at each loci leads to the interpretation of the standardized score as a *common statistical score function* based on the derivative of a corresponding likelihood function (see Kong and Cox, 1997).^18^

However, note that although the choice of test statistic and possible standardization procedure are important from a testing and statistical significance perspective it is not particularily essential for the present discussion. Moreover, generally the interpretations and relative performances of the different score function variants will not change when dealing with imperfect data, hence this matter is only noted on in this specific subsection.

### 2.6 Two-locus score functions

One may generalize the one-locus procedure above in order to simultaneously, or sequentially, search for two distinct disease loci on the genome. The former case is referred to as an *unconditional* analysis, whereas the latter case is a *conditional* analysis performed conditioning on some kind of genetic information at one, or several, conditioning loci. One may generally use the same basic score function definitions in both cases, taking into account that the standardizations will differ.

Implicitly defined score functions may in some cases be relatively easily generalized to the two-locus case, but in some cases the corresponding score-algorithm will be refrainingly more complex. As a positive example, one may generalize (5) into a two-locus score function. In Ängquist et al. (2007) the following, quite general, formulation is given

(13) $$S_{\text{pairs}}(w_{1},w_{2})=\sum_{i<j}{\left[{\text{IBD}}_{i,j}(w_{1})+{\text{IBD}}_{i,j}(w_{2})\right]}^{k},$$

where IBD*_i,j_* (*w_i_*) equals IBD(*a_i_, a_j_*) in (5) with respect to inheritance vector *w_i_*, related to the *i*th (disease or marker) loci, and {*a_i_*, *a_j_*} ∈ 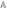.

For *k* > 1 (13) may be thought of as trying to capture epistatic joint pairwise IBD-sharing within a pedigree. The case *k* = 1 of (13) corresponds to the additive score function used in Strauch et al. (2000),

$$\begin{array}{l} S_{pairs}(w_{1},w_{2})=\sum_{i<j}\left[{\text{IBD}}_{i,j}(w_{1})+{\text{IBD}}_{i,j}(w_{2})\right]\\ =\sum_{i<j}{\text{IBD}}_{i,j}(w_{1})+\sum_{i<j}{\text{IBD}}_{i,j}(w_{2})\\ =S_{\text{pairs}}(w_{1})+S_{\text{pairs}}(w_{2}), \end{array}$$

which these authors also implemented into the analysis program GENEHUNTER-TWOLOCUS. In the applications of Ängquist et al. (2007) the case *k* = 2 is used, which shows close to NCP-optimal performance for the one-parameter genetic disease model families used in their simulations.

#### Example 8 (ASP score matrix)

*For ASPs one might summarize a two-locus score function completely using a* 3 × 3 *score matrix.*^19^ *Letting*

$$S(i,j)=S({IBD}_{1}=i,{IBD}_{2}=j),$$

where IBD_k_ = l means that the ASP shares l alleles IBD at the kth (marker or disease) locus, leads to the general score matrix

(14) $$S=\left[\begin{array}{ccc} S(0,0) & S(0,1) & S(0,2)\\ S(1,0) & S(1,1) & S(1,2)\\ S(2,0) & S(2,1) & S(2,2) \end{array}\right]$$

*Several instances and substructures of (14) are given, implemented and discussed in* *Ängquist et al.* (2005).

Two-locus explicitly defined score functions are concept-wise straightforward generalizations of one-locus ones. Moreover, the NCP-optimal score function (10) of Ängquist et al. (2007), for unconditional and conditional two-locus analysis respectively, may be generalized to

(15) $$\{\begin{array}{l} S(w_{1},w_{2})\propto P(w_{1},w_{2})-2^{-2m},\\ S(w_{1}|w_{2})\propto P(w_{1}|w_{2})-2^{-m}. \end{array}$$

Note that the interpretation of these scores as being proportional to probability-based differences with respect to the null and (assumed) alternative hypotheses still hold true.

## 3 Generalization to the Optimality-Based Definition Approach

In some cases where the true disease model *λ* is fully or partially unknown the usage of the NCP-optimal score function (10) based on an estimate (or assumption) *λ̂* may be considered to lack robustness and applicability. In order to reduce unnecessary usage-avoidance we will next try to further generalize this approach, hence increasing its practical usefulness. More explicitly, our suggested method is adapted to include prior, assumed or intuitive, information on *λ* in a more direct sense than what is available through using explicit versions, but still avoiding the assumption of a *single* plausible genetic disease model.

### 3.1 Algorithm

Begin with choosing *d* distinct genetic disease models

$$\{\lambda_{1},\lambda_{2},\ldots ,\lambda_{d}\}\in \Lambda ,$$

with inheritance distributions under corresponding alternatives

$$P_{i}=P(w|\lambda_{i});i=1,2,\ldots ,d.$$

Now, a simple generalization to the previous score in (10) is given by

(16) $$S(w)\propto \frac{\sum_{i=1}^{d}\left[P(w|\lambda_{i})-2^{-m}\right]}{d},$$

where *d* in the denominator in principle is unnecessary (according to the standardization) but makes comparisons between (10) and (16) possible in a natural way.

A further generalization arises if adopting a *Bayesian perspective* with respect to the prior distribution of possible disease models.^20^ Fix *d* and let π = (π_1_, π_2_,…,π*_d_*), with ∑*_i_*_=1_*^d^* *π_i_* =1, be the vector of prior probabilities corresponding to the *d* distinct disease models. This leads to (16) being generalized into

(17) $$S(w)\propto \sum_{i=1}^{d}\pi_{i}[P(w|\lambda_{i})-2^{-m}].$$

One may note that (16) is the special case of (17) where π = (1/*d*, 1/*d*,…,1/*d*) and that (10) correspond to *d* = 1 and hence π = π_1_ = 1 for a single disease model *λ*_1_. Finally, observe that the NCP-optimality property (Ängquist et al. 2007) is kept if (in a somewhat abstract sense) π, given the present knowledge-base, is the true probability distribution with respect to the present genetic disease model-ambiguity.

## 4 A Small Simulation Study

**Figure 4:**
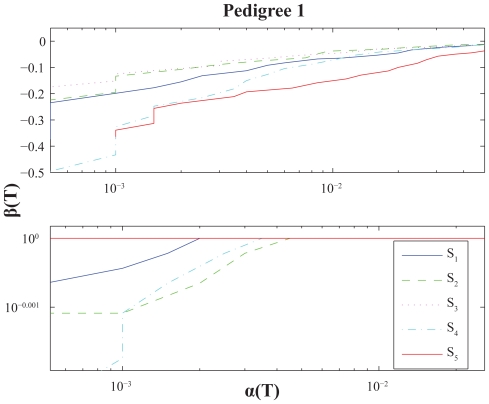
Power calculations for Pedigree 1 and score functions *S*_1_-*S*_5_. Presented as ROC-curves with significance levels α(*T*) vs. powers β(*T*) for score thresholds *T*. (Logarithmic X/Y-scales.) Upper and lower panel uses penetrance vectors *f* = (0.02, 0.2, 0.8) and *f* = (0.02, 0.8, 0.8) respectively.

**Figure 5:**
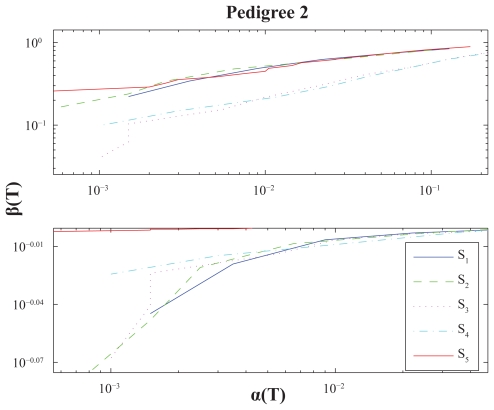
Power calculations for Pedigree 2. See caption of Figure 4.

**Figure 6:**
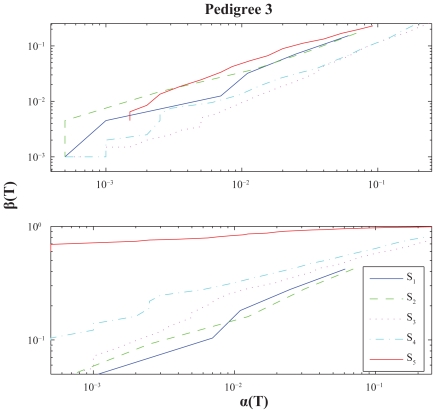
Power calculations for Pedigree 3. See caption of Figure 4.

For illustrational purposes we include a small-scale simulation analyses in this section. We perform power calculations for various settings and present them through *ROC-curves*, i.e. as plots with significance levels versus power with respect to a set of underlying score thresholds (Selin, 1965; Bradley, 1996). The results are given, and graphically displayed, in Figures 4–6.

### 4.1 Simulation set-up

Consider a pedigree consisting of two parents of unknown phenotypes and *M* siblings. For instance, Pedigree 3 in Figure 1 is such a pedigree with *M* = 5. We construct three *homogeneous* pedigree sets, i.e. a set consisting of pedigrees with similar structure and phenotype setting only, based on three distinct such pedigrees with *M* = 6: (i) Pedigree 1 consisting of 4 affected and 2 unaffected siblings. (ii) Pedigree 2 consisting of 3 affected and 3 unaffected siblings. (iii) Pedigree 3 consisting of 2 affected and 4 unaffected siblings. The number of pedigrees in each pedigree set is put to *N* = 15.

Further, for each case we use a genome consisting of a single chromosome of length *G* = 4 Morgans, *J* = 2000 simulations and score thresholds ranging from *T* = 3 to *T* = 10. The analyses are made with respect to five distinct score functions: The *S*_1_ = *S*_pairs_ function in (5), the *S*_2_ = *S*_all_ function in (6), the extended version, *S*_3_, using (7) for *S*_pairs_, the extended version, *S*_4_, using (7) for *S*_all_, the NCP-optimal score function *S*_5_ in (10). All score functions are standardized through (11) and calculations are performed using the NPL score approach (12).

Finally, we used two genetic models, *λ*_1_ and *λ*_2_, where both correspond to disease allele frequency *p* = 0.01, but with distinct penetrance vectors,

$$\begin{array}{l} f=(f_{0},f_{1},f_{2})=(0.02,0.20,0.80)\;\text{and}\\ \left(0.02,0.80,0.80\right) \end{array}$$

respectively. Here *f_i_* denotes the probability for an individual, having a disease genotype consisting of *i* disease alleles and 2 − *i* normal alleles, of being affected.

### 4.2 Results and discussion

It is quite hard to draw very certain conclusions from such a small study, once more note that this section is in some sense a side-track, but a few general observations of some interest may be stated: (i) *S*_2_ performs better than *S*_1_ for Pedigree 1, whereas the opposite is true for Pedigree 2–3 under *λ*_2_. In other words their relative performance is affected by the pedigree structure as noted above. (ii) The extended versions *S*_3_ and *S*_4_ often outperforms the traditional (nonextended) versions *S*_1_ and *S*_2_. These extensions seem somewhat more favourable for Pedigree 3 than for Pedigree 1, which seems reasonable since the latter pedigree has a structure more directed towards unaffected individuals. They also seem more advantageous under *λ*_2_ than for *λ*_1_, which might be explained by the latter model having more IBD-sharing discrimination power within the subgroup of unaffecteds; according to a higher disease penetrance for disease heterozygotes. (iii) The NCP-optimal score function *S*_5_ is performance-wise much better under *λ*_2_. Probably mainly follows from similar reasoning as given in the last sentence under (ii).

## Footnotes

1 A *nonfounder* (*founder*) has both (has not any) of its parents included in the pedigree. The rationale for the number of meioses being *m* = 2(*n* − *f*) is that, which follows from above, each of the *n* − *f* nonfounders corresponds to two meioses. Here *n* and *f* are the total number of individuals and the number of founders in the pedigree respectively.

2 In other words, they are both inherited instances of *g**_i_* ∈ , for some *i* = 1, 2, …, 2*f*, where  is the set of all 2*f* founder alleles and *f* is the number of founders.

3 One may note that this notion of a score function may be seen as adopting a data-mining perspective where such functions are used for scoring patterns (Hand et al. 2001). In this case one observes and scores inheritance patterns.

4 Or more generally within phenotype-groups.

5 For instance using the standard decimal interpretation (conversion) of the binary zero-one inheritance vector with length *m*.

6 According to the fact that the pedigree construction excluded farther (earlier) generations.

7 Including the ordered affected individuals *a*_1_, *a*_2_,…,*a*_| |_, where | | is the number of affecteds or, equivalently, the *cardinality* of the set .

8 Each specific selection *h* consists of | | alleles that may be grouped according to their ancestral history, i.e. each allele is a copy of one of the 2*f* founder alleles. The link to the number of members in the *i*th group is *b**_i_*(*h*), i.e. where *b**_i_*(*h*) is the number of *g**_i_* alleles; see Footnote 2.

9 Generally *S* (*v*| ) means applying the traditional score function *S*, given *v*, to the arbitrary pedigree subgroup . Also note that  ∪   equals the subgroup of all individuals in the pedigree with *known* phenotype.

10 This might be the case for, in some relative (to the disease) sense, *young* individuals. These persons might be interpreted as what we later refer to as *ambiguous* cases.

11 Note that *P*(*w*|*H*_0_) = 2^−^*^m^* for all *w* ∈ .

12 Note that we end up with the standardized properties *E*(*S*|*H*_0_) = 0 and *V*(*S*|*H*_0_) = 1.

13 Two score functions *S*^1^ and *S*^2^ (unstandardized or standardized) are equal if, using the formulation of (3), *s**_i_*^1^ = *s**_i_*^2^ for all *i*.

14 This follows since these settings uniquely define the standardization parameters *μ* and *σ* in (11).

15 In other words, when the complete inheritance process over corresponding loci is not known with probability one.

16 Note that (12) refer to a single pedigree (*pedigree-specific NPL score*; see Ängquist (2007) for more information). For a full pedigree set one uses pedigree-weighted sums with respect to such present scores.

17 A textbook on HMMs is Cappé et al. (2005).

18 On *standard score functions* see e.g. Clayton and Hills (1993) or Garthwaite et al. (1995). A specialized monograph on the theory and philosophy of the *likelihood approach* is Edwards (1992).

19 Note that this is possible according to our assumption of scoring all inheritance vectors leading to similar IBD-sharing structures equally. In this case, at each locus, the *three* distinct IBD-sharing structures correspond to the number of alleles (0, 1 or 2) shared IBD by the affected sib-pair.

20 Subjective or empirically objective perspective; for concepts see e.g. Winkler (1972) and Gelman et al. (2004).

## Appendix Some Technicalities

### A Equivalence regarding IBD-sharing Structures

Vaguely speaking equivalent IBD-sharing structures means that these structures correspond to structurally similar (exchangeable) genetic inheritance. Consider two structures, *A* and *B* say. In its simplest form this above property correspond to cases where *A* is transformed to *B* if the inheritances of two individuals, of similar pedigree structure positions, are switched (permuted).

#### Example 9 (Equivalent AST-structures)

*Assume an affected sib-trio (AST) pedigree as shown in *Figure 2. Order the three affected siblings as Sibling 1, 2 and 3 respectively. Quite naturally, let IBD(i, j) = k mean that the ith and jth sibling share k alleles IBD.^21^

*If IBD*(1, 2) = 2 *and IBD*(1, 3) = 0, *implicitly IBD*(2, 3) = 0.^22^ *Now, permute Sibling 1 and Sibling 3. This leads to IBD*(1, 2) = 0 *and IBD*(1, 3) = 0, *and IBD*(2, 3) = 2. *These two cases, i.e. the corresponding inheritance vectors, are assumed to produce equivalent IBD-sharing structures.*

### B Criterion regarding unstandardized score function equivalence

Formulating criterions for producing equivalent (equivalence classes of) score functions turns out to be most straightforwardly achieved using the general score function definition (3).

When *n* = 2, given an underlying score distribution under *H*_0_, there is only one type of standardized score function definition, i.e. regardless of the instantiation of the unstandardized scores, *s*_1_ and *s*_2_, one ends up with the same standardized function. Explicitly, all score functions are equivalent.

In the same manner, when *n* = 3, for each value (constant) *c* ∈ [0, ∞] the relation

(18) $$s_{3}-s_{2}=c(s_{2}-s_{1})$$

defines a single specific equivalence class.^23^ The meaning of this is that all unstandardized score functions fulfilling the above relation with the same *c* are forced to become equivalent.

#### Example 10 (Equivalence class of *S*_pairs_ and *S*_all_)

*For an ASP, as noted above, S**_pairs_* *and S**_all_* *are equivalent. The whole corresponding equivalence class, including these two instances, are defined through using c =* 1 *in (18). This class is constituted by all symmetric score functions, i.e. such functions obeying s*_3_ − *s*_2_ = *s*_2_ − *s*_1_.

Finally consider an arbitrary *n* > 3. Here, each ordered vector of constants (*c*_1_, c_2_, …,*c**_n_*_−1_) where all *c**_k_* ∈ [0, 1], with natural restricting constraint ∑*_k_*_=1_*^n^*^−1^*c**_k_* = 1, and the vector moreover fulfills

$$\left[\frac{s_{k+1}-s_{k}}{s_{n}-s_{1}}\right]=c_{k};\;k=1,2,\ldots ,n-1,$$

then defines an equivalence class of (unstandardized) score functions in the same sense as above.
